# MRI-based static and functional assessment of complex hip deformities in comparison with CT: a validation study

**DOI:** 10.1007/s10334-020-00845-5

**Published:** 2020-04-22

**Authors:** Sophia Blum, Lea Franken, Albrecht Hartmann, Falk Thielemann, Verena Plodeck, Dirk Danowski, Jens-Peter Kühn, Ralf-Thorsten Hoffmann, Klaus-Peter Günther, Jens Goronzy

**Affiliations:** 1 Department of Radiology, Institute and Policlinic of Diagnostic and Interventional Radiology, University Hospital Carl Gustav Carus, Technische Universität Dresden, Dresden, Germany; 2 University Center of Orthopedics and Traumatology, University Hospital Carl Gustav Carus, Technische Universität Dresden, Dresden, Germany

**Keywords:** Virtual ROM measurements, Hip deformity, Automated ROM measurement, Femoroacetabular impingement, Alpha angle, Magnetic resonance imaging

## Abstract

**Objective:**

This study aimed at investigating the agreement between predefined quantitative parameters of hip morphology derived from magnetic resonance imaging (MRI) and virtual range of motion (ROM) analysis using computed tomography (CT) as standard of reference.

**Methods:**

Twenty patients (13 females, 7 males, 16–59 years) with hip deformities underwent MRI prior to surgery. Clockwise alpha angle, femoral head and neck diameter, collum caput diaphysis angle, femoral torsion, center-edge angles, acetabular coverage of the femoral head, sourcil angle, and acetabular anteversion were measured. Additionally, tern single and combined movements were simulated using a motion analysis program. The MRI findings were compared with the corresponding results obtained by CT. Correlation of MRI with CT was assessed using different statistical methods (intraclass correlation coefficient, Bland–Altmann plot, two one-sided *t *test), and linear regression analysis was performed.

**Results:**

The results showed near-perfect intraclass correlation coefficients (ICCs) for anteversion (0.95), acetabular sector angles (0.98–0.99), sourcil angle (0.95), and acetabular coverage (anterior 0.96, posterior 0.99). Intermethod correlation for femoral parameters showed almost perfect agreement except for the alpha angle (0.73–0.97). No significant proportional bias was detected for traditional acetabular and femoral parameters. ROM analysis was performed for 370 movements in 37 hips. For 78.4% (290/370) of the movements analysed, neither CT nor MRI detected impingement across the physiological ROM. For 18.6% (69/370) of the movements, impingement was detected by both CT and MRI, while 2.2% (8/370) of the movements with impingement in CT showed no impingement in MRI, and 0.8% (3/370) of the movements with impingement in MRI had no corresponding result in CT.

**Conclusion:**

Finally, it was concluded that MRI-based assessment of hip morphology and virtual ROM analysis is feasible and can be performed with good intermethod agreement in comparison to the gold standard (CT). Therefore, MRI appears to be substantially equivalent to CT for use in virtual ROM analysis and so may reasonably be used in place of CT for this purpose.

## Introduction

Our understanding of hip pathobiomechanics has evolved over the past decades and has led to advances in joint-preserving surgery [1]. Accurate analysis of hip morphology by advanced imaging modalities had an important role in this development [1]. Besides determination of conventional radiographic parameters, radiologists can use computer-assisted animation to display the virtual range of motion (ROM) and detect possible sources of intra- and extra-articular impingement [2–4]. This information helps the surgeon to better understand the deformity, especially in complex cases with combined deformities, and plan the best surgical strategy [1]. Over the last decade, the technique for motion analysis has improved from using a fixed predetermined center of rotation to the computation of an acetabular and femoral sphere, which maintains a dynamic center of rotation with an equidistant joint space and thus makes virtual ROM more precise [3]. The algorithms for virtual ROM analysis have been validated on cadaveric models by comparing actual and virtual ROM based on computed tomography (CT) data [2, 5].

Previously, only CT was used for virtual ROM measurement due to its superior contrast between bone and soft tissue and spatial resolution. Despite the radiation hazard, CT continues to be regarded as the gold standard for quantifying bone abnormalities [6]. Therefore, reliable alternative imaging techniques not using ionizing radiation are desirable, especially as many of the patients requiring hip-preserving surgery are children or young adults. State-of-the-art high-resolution 3D magnetic resonance imaging (MRI) sequences fulfill these requirements and can be used to acquire imaging data for virtual motion analysis. MRI has been shown to be comparable to CT for the static measurement of the femur [7] and acetabulum [8, 9]. With the advent of improved semiautomatic segmentation algorithms for volumetric MRI datasets, it has become possible to use virtual ROM analysis in clinical practice [4]. Therefore, the purpose of this study was to assess the feasibility of MRI-derived quantification of hip joint morphology and virtual ROM analysis in patients with hip deformities using CT as a standard of reference.

## Methods

### Patients

The study was approved by the local ethics committee (EK 531122015). Twenty consecutive patients (13 females, 7 males) with hip deformities scheduled for surgery were enrolled. They had a mean age of 32.2 (16–59) years. The underlying pathology was isolated femoroacetabular impingement (FAI) in 17 cases and combined dysplasia plus FAI in three cases. None of the patients had prior pelvic or hip surgery. For the study, motion analysis was performed on both the affected joint and the contralateral joint. One patient was excluded because of incomplete depiction of the pelvis. In another case, only one hip could be assessed due to motion artifacts of the knee. Therefore, imaging data of 37 hips in 19 patients were available for analysis.

### Imaging and data analysis

The patients included underwent a clinically indicated CT examination for motion analysis and were additionally examined by MRI. Areas scanned using CT and MRI included the pelvis, covering the anterior superior iliac spine (ASIS) and the lesser trochanter, and another scan across the femoral condyles.

CT examinations were performed on a Siemens Somatom Definition AS + (Erlangen, Germany) with a reconstructed slice thickness of 0.6 mm (0.8 mm pixel spacing). MRI was performed in a 3-T Siemens Magnetom Verio (Erlangen, Germany) using a 3D isotropic volume-interpolated breath-hold examination (VIBE) sequence with 0.8 mm slice thickness for high spatial resolution (TE 4.9 ms, TR 10.8 ms, and 6.33 min acquisition time) and a T2-weighted half-Fourier acquisition single-shot turbo spin echo (HASTE) sequence of the knees (TE 101 ms, TR 1000 ms, and 0.67 s acquisition time). The T2-weighted HASTE sequence was chosen because it has a very short acquisition time and provides sufficient slice thickness (5 mm) to delineate the femoral condyle. For both CT and MRI, the patients’ legs were positioned in internal rotation with a pillow underneath the popliteal fossa. During MRI, the legs were additionally kept in position by sandbags. The study patients were examined with an extended routine MRI protocol with the VIBE sequence starting at the ASIS instead of at the supra-acetabular region. The pulse sequences were acquired in a preset chronological order starting with T2 HASTE, then VIBE, and then other sequences. When deemed necessary, the technician could switch the order of pulse sequences so that the VIBE and HASTE sequences were not acquired consecutively in each patient.

Both MRI and CT datasets were analysed using the Move Forward ™ software from Clinical Graphics (Delft, Netherland; Zimmer Biomet). The Clinical Graphics tool is based on a semiautomatic segmentation procedure as described in detail by Röling et al. [2] that processes the complete MRI and CT datasets and issues standardized reports of the results. Virtual motion analysis was performed in a standardized pelvic orientation accomplished by angling the anterior pelvic plane (APP) using the ASIS on both sides and the pubic tubercle, and then adding an anterior tilt of 3°. The femoral plane for motion analysis was then established by locating the femoral head center, the midpoint of the two epicondyles, and the epicondylar axis at the knee. The virtual motion analysis software identified impingement where there was at least a 3-mm translational displacement of the femoral head relative to the acetabulum. Soft tissue was not considered in ROM analysis. In addition, the software routinely measures the following conventional radiological parameters for the femur: clockwise alpha angle from 9 to 3 o’clock, femoral head and neck diameter (in mm), collum caput diaphysis angle (CCD angle), and femoral torsion. For the acetabulum, the following parameters were measured: center edge angle at 1, 12, and 11 o’clock (acetabular sector angle—ASA), anterior and posterior acetabular coverage of the femoral head in percent, as described by Dandachli et al. [10], sourcil angle, and acetabular anteversion at the level of the joint center and in the midpoint between joint center and acetabular roof.

For virtual ROM analysis, the software simulates ten different single-plane and combined movements using the CT and MRI datasets. First, isolated flexion, abduction, and internal rotation are simulated. Combined movements include internal or external rotation and 50° abduction, internal rotation with 90° flexion, extension with 15° external rotation, and internal rotation with 20° adduction and 30°, 60° and 90° flexion. With the software used, motion and therefore, also impingement is only identified within predefined physiological ranges, as provided in Table 1 for each of the movements analysed. If the simulated ROM was not in the physiological range as defined by the software manufacturer, the ROM angle (pathological virtual mechanical conflict) was determined in both MRI- and CT-based analysis.

**Table 1:** Results of simulated range of motion in CT and MRI

Movement		Maximum movement (°)	No impingement in either CT or MRI	Impingement in CT only	Impingement in MRI only	Discordant result	Mean absolute difference (°) (min–max)
Flexion	Isolated	120	24	13	10	3	− 0.32 ± 3.49 (− 14 to 9)
Extension	15° of external rotation	15	29	8	6	2	− 0.30 ± 1.05 (− 5 to 1)
Abduction	Isolated	50	32	3	5	2	0.70 ± 3.67 ( 3 to 21)
Internal rotation	Isolated	50	37	0	0	0	0 ± 0 (0)
	50° abduction	40	29	8	7	1	− 0.65 ± 2.84 (− 15 to 0)
	90° flexion	30	26	11	10	1	− 0.62 ± 2.83 (− 12 to 5)
	30° flexion, 20° adduction	50	37	0	0	0	0 ± 0 (0)
	60° flexion, 20° adduction	40	30	7	7	0	0.22 ± 2.44 (− 5 to 13)
	90° flexion, 20° adduction	30	17	20	19	1	− 0.35 ± 2.31 (− 6 to 9)
External rotation	50° abduction	40	29	7	8	1	0.03 ± 4.21 (− 21 to 12)

Displayed are the maximum simulated motion for the movements analysed, the absolute number of movements with simulated impingement in CT and MRI, absolute number of cases with discrepant results in CT and MRI, and mean difference in range of motion between CT and MRI (mean ± standard deviation [minimum—maximum])

All reports were reassessed by visual analysis for correct position of orientation points in either CT or MRI by two radiologists (SB and JG). Incorrect positioning was recorded when the orientation points used by the software tool were not in the expected position relative to the cortical bone.

### Statistics

SPSS^©^ 23 (IBM) and RStudio Version 3.2.3 (package: equivalence) were used for statistical analysis. The data was analysed for agreement. To assess the strength of agreement, intraclass correlation coefficients (ICC) were calculated for each radiological parameter. A two-way mixed model with absolute agreement was used. Coefficients were interpreted as follows: ICC < 0.2 “slight agreement”; 0.2 ≤ ICC < 0.4 “fair agreement”; 0.4 ≤ ICC < 0.6 “moderate agreement”; 0.61 ≤ ICC < 0.8 “substantial agreement”; and ICC ≥ 0.8 “almost perfect agreement” [11]. To test for equivalence of MRI and CT a two one-sided *t* test (TOST) with an *Ɛ* (magnitude of region of similarity) of 2° and a level of significance of 5% was performed. TOST-derived *p* values were corrected for multiple comparisons using the Bonferron–Holm method. Bland–Altman plots were prepared for all measurements to assess the agreement of MRI in comparison to the reference standard CT. For further interpretation of the Bland–Altman plots and to detect proportional bias, a linear regression procedure with the difference between two groups as the dependent variable and mean overall difference as the independent variable was performed. As patients may move their legs between the VIBE and HASTE sequences, the time elapsed between the two was recorded. To identify the possible influence of this time span on measuring inaccuracy a chi-square test was performed. For femoral torsion and all parameters determined by ROM analysis a deviation > 3° was defined to indicate a surgically relevant finding. To minimise leg movement, the interval between VIBE and HASTE sequences should be ≤ 5 min.

## Results

**Fig. 1:**
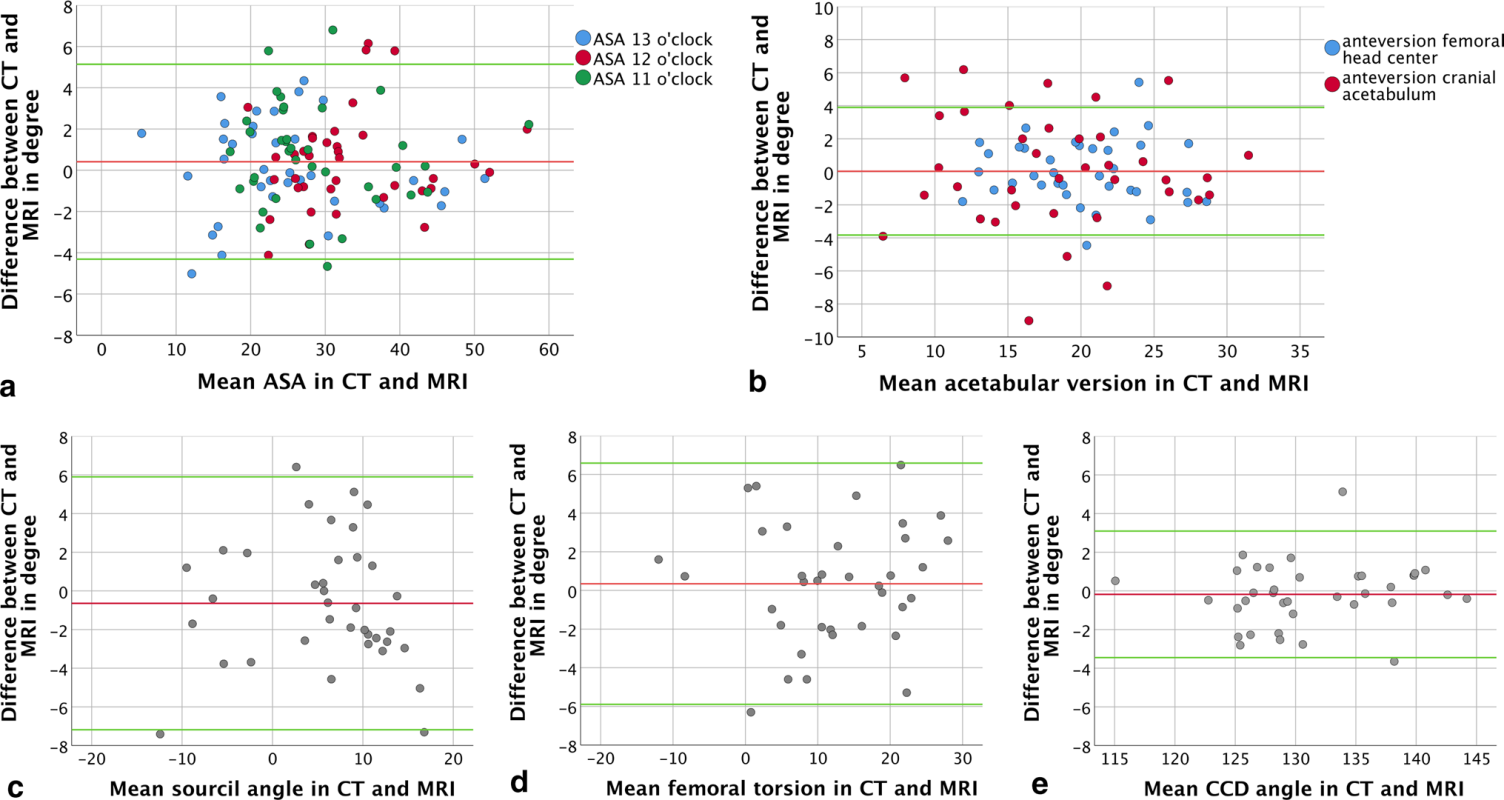
Bland–Altman plots of differences between MRI- and CT-derived measurements; red line—mean; green line—1.96 standard deviation. **a** Mean acetabular sector angle (ASA) (green—ASA 11 o’clock, red—ASA 12 o’clock, blue—ASA 13 o’clock), **b** Mean acetabular version (blue—version at the level of femoral center; red—version in the cranial aspect of the acetabulum). **c** Mean sourcil angle, **d** femoral torsion, **e** caput collum diaphysis (CCD) angle

Acetabular parameters showed excellent ICCs for intermethod correlation with “almost perfect” agreement for anteversion, ASA, sourcil angle, and acetabular coverage (Table 2). The Bland–Altman plots revealed an even distribution of differences around the zero line with 95% confidence intervals (95% CIs) of 0.08° ± 3.46°, − 0.64° ± 3.27°, and up to 0.40° ± 2.36° for acetabular anteversion, sourcil angle, and acetabular sector angles, respectively (Fig. 1a–c). Linear regression analysis detected no significant proportional bias, and the TOST showed no significant occurrence of mean differences ≥ 2° for any of the comparisons (Table 2).

**Table 2:** Intermethod analysis with mean difference [mean ± standard deviation (minimum–maximum)], *p* value of two one-sided *t *test (TOST; *α* = 0.05, *Ɛ* = 2°), linear regression analysis with the difference between MRI and CT as the dependent variable and the mean as the independent variable (regression coefficient [*p*]), and correlation analysis (intraclass correlation coefficient [95% confidence interval])

	Parameter	Mean absolute difference (min–max)	P (TOST)	Linear regression Regression coefficient (*p*)	Intraclass correlation coefficient ICC (95% confidence interval)
Acetabular version	Femoral head center	0.08 ± 3.46 (− 4.4 to 5.4)	0.001	− 0.023 (0.76)	0.953 (0.909–0.976)
	Mid point	0.04 ± 1.93 (− 9.0 to 6.2)	0.001	− 0.35 (0.57)	0.936 (0.898–0.960)
Acetabular sector angle	11	0.69 ± 2.50 (− 4.7 to 6.8)	0.001	− 0.017 (0.72)	0.979 (0.959–0.989)
	12	0.40 ± 2.36 (− 4.1 to 6.2)	0.001	0.034 (0.46)	0.982 (0.965–0.991)
	13	0.15 ± 2.27 (− 5.0 to 4.3)	0.001	− 0.015 (0.67)	0.989 (0.979–0.995)
Sourcil angle		− 0.64 ± 3.27 (− 7.4 to 6.4)	0.001	− 0.026 (0.73)	0.953 (0.909–0.976)
Acetabular coverage	Anterior	0.69 ± 1.42 (− 2.1 to 4.2)	0.001	− 0.019 (0.75)	0.964 (0.914–0.983)
	Posterior	0.41 ± 1.1 (− 2.1 to 3.1)	0.001	− 0.027 (0.44)	0.988 (0.975–0.994)
Alpha angle	9	1.3 ± 4.05 (− 8.4 to 10.42)	0.03	− 0.030 (0.86)	0.729 (0.467–0.860)
	10	− 0.14 ± 3.94 (− 8.4 to 7.6)	0.001	− 0.149 (0.19)	0.886 (0.778–0.941)
	11	− 1.17 ± 3.00 (− 7.3 to 3.8)	0.001	− 0.075 (0.50)	0.882 (0.758–0.941)
	12	− 2.50 ± 4.10 (− 13.2 to 6.0)	0.23	0.084 (0.33)	0.916 (0.765–0.963)
	13	− 1.42 ± 4.25 (− 12.6 to 8.5)	0.03	− 0.016 (0.79)	0.967 (0.933–0.983)
	14	0.76 ± 3.69 (− 6.6 to 7.8)	0.001	0.066 (0.36)	0.954 (0.911–0.976)
	15	0.69 ± 4.43 (− 7.6 to 12.8)	0.02	− 0.020 (0.84)	0.919 (0.843–0.958)
Femoral torsion		0.35 ± 3.12 (− 6.3 to 6.5)	0.001	0.028 (0.62)	0.974 (0.949–0.987)
Caput collum-diaphysis angle		− 0.18 ± 1.64 (− 3.7 to 5.1)	0.001	− 0.032 (0.46)	0.984 (0.969–0.992)
Diameter	Acetabular	– 0.97 ± 1.8 (− 15.3 to 4.3)	0.001	− 0.216 (0.93)	0.844 (0.690–0.921)
	Femoral head	0.23 ± 0.57 (− 1.1 to 1.1)	0.001	− 0.024 (0.36)	0.993 (0.985–0.997)
	Femoral neck	0.44 ± 0.55 (− 0.5 to 1.9)	0.001	0.002 (0.92)	0.991 (0.958–0.997)

**Fig. 2:**
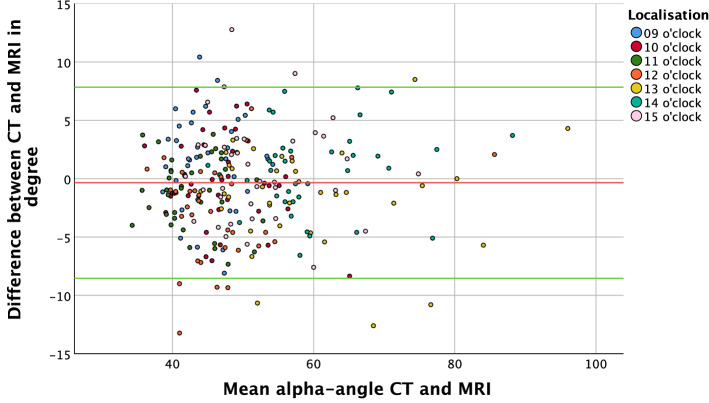
Bland–Altman plot of difference between MRI and CT in the measurement of alpha angles; red line—mean; green line 1.96 standard deviation (blue—9 o’clock; red—10 o’clock; green—11 o’clock, orange—12 o’clock, yellow—13 o’clock, turquoise—14 o’clock, pink—15 o’clock)

Femoral parameters showed good correlation as well. However, with a difference ranging from − 2.5° to 1.3° the comparability of the alpha angle was not as good as that of the other parameters. Still, all ICCs for intermethod correlation showed almost perfect matching except for the alpha angle at 9 o’clock, which demonstrated only ‘substantial agreement’ (Table 2). The Bland–Altman plot also revealed a wider 95% CI and an even distribution around the zero line (Fig. 2). The TOST showed no significant occurrences of  ≥ 2° differences for the alpha angle, except at 12 o’clock (Table 2).

**Fig. 3:**
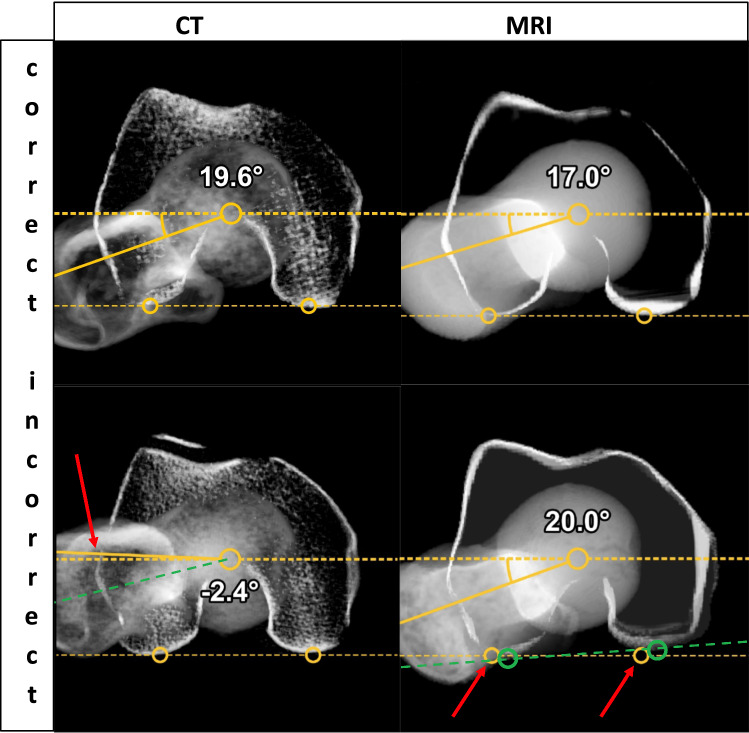
Example of correct (upper row) and incorrect positioning (lower row, red arrows) of markers for measurement of femoral torsion (with green lines and circles indicating correct positions in the lower row)

**Fig. 4:**
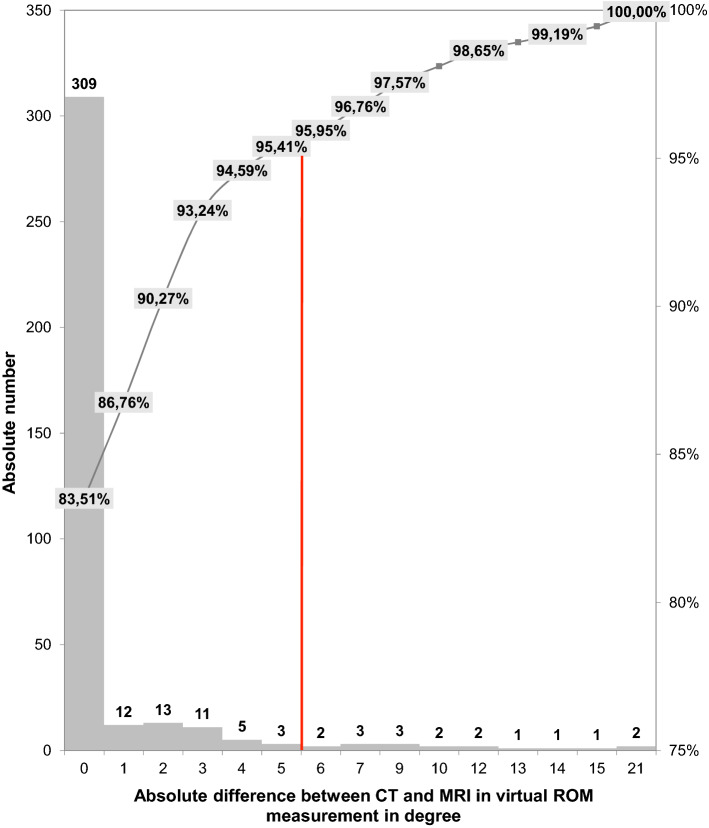
Histogram showing the distribution of absolute differences between MRI- and CT-derived virtual ROM measurements. The red dashed line indicates a 5° difference

For femoral torsion and the CCD angle, the mean absolute difference was 0.35° ± 3.12° and − 0.18° ± 1.64°, respectively. The Bland–Altmann plots are shown in Fig. 1d, e. An ‘almost perfect’ intermethod agreement was found for femoral torsion (ICC 0.97) and CCD angle (ICC 0.98) (Table 2). Detailed analysis of the cases with differences greater than 3° in femoral torsion revealed an incorrect position of orientation points in either CT or MRI in 4 of 37 hips. To illustrate, two cases are presented in Fig. 3. Linear regression analysis detected no significant proportional bias (Table 2). No significant influence of a time interval  > 5 min between VIBE and HASTE sequences on discordant results between MRI and CT was found for femoral torsion (*p* = 0.91, Table 3) (Fig. 4).

**Table 3:** Contingency table displaying the frequency distribution of discrepant results between MRI and CT for torsion measurement (defined as difference in torsion ≥ 3°) in MRI examinations with intervals ≤ 5 min between HASTE and VIBE sequences versus examinations with intervals > 5 min

Discordant result between MRI and CT per hip	Interval between HASTE and VIBE ≤ 5 min	Interval between HASTE and VIBE > 5 min	Total	*p *value (Chi-square statistic)
No	8	16	24	0.8736
Yes	4	9	13	
Total	12	25	37

**Fig. 5:**
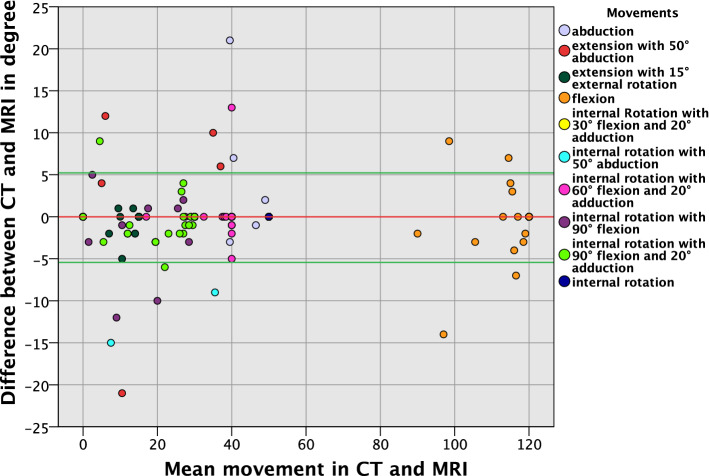
Bland––Altman plot for all ROM analyses displaying differences between MRI- and CT-derived measurements; red line—mean; green line—1.96 standard deviation (light blue—abduction; red—extension with 50° abduction; dark green—extension with 15° external rotation, orange—flexion, yellow—internal rotation with 30° flexion and 20° adduction, turquoise—internal rotation with 50° abduction, pink—internal rotation with 60° flexion and 20° adduction, violet—internal rotation with 90° flexion, light green—internal rotation with 90° flexion and 20° adduction, dark blue—internal rotation)

A total of 370 ROM analyses were performed in 37 hips (10 different movements per hip). In 78.4% (290/370) of the analysed movements, neither CT nor MRI detected impingement in the predefined physiological range of motion (Table 1). In 18.6% (69/370) of the movement analyses, both CT and MRI detected impingement in the physiological range of motion with a mean difference of − 0.1° ± 2.4° (− 21° to 13°). In 2.2% (8/370) of cases, only CT motion analysis, but not MRI analysis, detected impingement in the physiological range of motion. In 0.8% (3/370) of cases, impingement was detected by MRI, but not by CT (Table 1). Bland–Altman plots revealed an even distribution of angles above and below the zero line (Fig. 5). A maximum mean absolute difference of 0.70° ± 3.67° was calculated (Table 1). 95% of the ROM analyses had a difference no larger than 5° and only 1.89% (7/370) showed a difference larger than 10°. Most differences occurred in the analysis of flexion (3/37), followed by extension, abduction, and external rotation (2/37). The analysis of internal rotation combined with other movements (especially flexion) showed only small differences between CT and MRI (3/222) (Table 1). An interval over 5 min between the VIBE and HASTE sequences was not associated with a higher rate of discordant results in ROM analysis between MRI and CT (*p* = 0.83, Table 4).

**Table 4:** Contingency table displaying the frequency distribution of discrepant results between MRI and CT for ROM measurement (defined as difference in ROM ≥ 3°) in MRI examinations with intervals ≤ 5 min between HASTE and VIBE sequences versus examinations with intervals > 5 min

Discordant ROM results between MRI and CT per hip	Interval between HASTE and VIBE ≤ 5 min	Interval between HASTE and VIBE > 5 min	Total	*p* value (Chi-square statistic)
No	6	15	21	0.8256
Yes	6	10	16	
Total	12	25	37	

## Discussion

The aim of this study was to investigate the agreement between MRI and CT in the diagnostic evaluation of hip morphology using predefined morphological parameters and virtual ROM analysis. It is the first study validating a novel MRI-based software tool for semiautomatic ROM analysis and conventional parameters of hip morphology compared to the gold standard, CT. The software used in this study was previously validated for CT [2]. Other studies already used MRI to analyse hip morphology, but none of them performed ROM analysis in comparison to CT [4, 12, 13].

The most important result of our study is that we found high correlation of radiological parameters and virtual ROM derived from MRI datasets with parameters derived from CT. There was no systematic bias for any of the measures as demonstrated by the Bland Altman plots and the intraclass correlations were 'almost perfect' (ICCs ≥ 0.8) for all radiological measures except the alpha angle at 9 o'clock.

Only a few studies have so far investigated semiautomatic or automatic segmentation of MRI datasets. These studies focused on static parameters and did not perform intermethod correlation. Goronzy et al. investigated manual measurements of acetabular anteversion and coverage of the femoral head and report mean absolute differences not exceeding 0.65° ± 0.86° for inter- and intraobserver as well as intermethod comparison [8]. Chu et al. compared manual and fully automatic segmentation of pelvic CT scans and found mean absolute differences of 2.0° ± 1.5° for acetabular version [14]. Moreover, they found a mean absolute difference of 3.5° ± 2.3° and a range of up to 10° for acetabular coverage. With the semiautomatic software tool investigated here, we found comparable results with a smaller range, suggesting that this type of segmentation is robust and that the results are reliable. Still, manual segmentation seems to be more precise than semiautomatic segmentation.

For ASA a very high intermethod correlation was shown (ICC: 0.98–0.99). The differences in ASA may be explained by the difficulty in differentiating between bone and labrum in MRI data, thereby leading to assumed over coverage. The mean differences of ASA were similar to the manual measurements performed by Goronzy et al. [8]. Exact measurement of the alpha angle can be demanding in both CT and MRI. Yan et al. compared manually measured alpha angles in CT and MRI, reporting a 95% CI up to 10° with an ICC between 0.60 and 0.78 [15]. Furthermore, they showed that agreement between CT and MRI depended on the underlying deformity, observing greater discrepancies for alpha angles in Cam deformities and acetabular parameters in dysplasia. Dessouky et al. report fair to moderate agreement for CT with manual segmentation in both interobserver analysis (ICC: 0.48–0.05) and intraobserver analysis (ICC: 0.49–0.24) [16]. At the same time Xia et al. present a patient collective in which they measured alpha angles in different MRI sequences with a fully automated software, as well as manually, and measured good ICCs (anterosuperior 0.84, anterior 0.92) [13]. The results Xia et al. report for the fully automated approach are slightly inferior to the results we obtained by semiautomatic measurement in the anterosuperior aspect of the femoral neck using CT and MRI (ICC: 0.92–0.97). Agreement for the alpha angle in the posterior part of the femoral head was still good but weaker (ICC: 0.73–0.89). Overall, the alpha angle seems more difficult to measure than other hip parameters such as acetabular version or coverage. Xia et al. found that this problem may be attributable to focal ossifications at the femoral head-neck junction, which may make it difficult to identify the contour of the femoral head, especially in MRI [13]. It may be for this reason that larger differences for the alpha angles in comparison to other pelvic measurements were found with 13 out of 259 cases (5%) exceeding 10∘ difference. Although results showed a good reliability using semiautomatic segmentation and measurements, the software may produce mistakes analysing irregular anatomy.

Although only 7 of the 370 virtual ROM analyses (1.98%) showed larger differences, they need to be discussed critically. Four of these measurements occurred in one patient. The HASTE images of this patient were degraded by motion artefacts distorting the bony outline of the femoral condyles. In this patient, not only the virtual ROM measurements but also femoral torsion measurements showed greater differences between MRI and CT. This case emphasizes the need for proper immobilisation of the legs during MRI and the importance of correlating virtually derived parameters with clinical findings. Not having excluded this case from analysis is a limitation of our study. At the same time, it is a good example of possible sources of error to be encountered when using this method in daily practice.

Regarding femoral torsion, we discovered small inaccuracies of the orientation points on either the femoral condyles or the femoral neck in both, CT and MRI (Fig. 3). Such inaccuracies are especially common for orientation points at the knee condyles due to the greater slice thickness of the HASTE sequence at this level. Botser et al. found higher interobserver reliability for CT than for MRI in manual measurements of femoral torsion. They also report that 77% of the cases had differences larger than 5° [7], whereas in our study only 14% of the cases exceeded this limit. Therefore, our results are superior to previous findings. Conversely, Tomczak et al. reported high intra- and interindividual reliability of femoral torsion measurements for both, CT and MRI [9]. Overall, these findings suggest that careful placement of orientation points by an experienced technician is desirable for both modalities. Additionally, the longer examination time using MRI may lead to leg movement between acquisition of different pulse sequences. In our study, we stabilised patients’ legs during the MRI examinations but VIBE and HASTE sequences were not always acquired consecutively, which is a possible drawback of our study. This can affect ROM analysis in general and parameters such as femoral torsion and increases the risk of motion artefacts. However, in our cohort, we did not find a relationship between longer intervals between the HASTE and VIBE sequences in our patients and outliers. Further, we did not detect a systematic relationship between discrepant torsion measurements and differences between MRI and CT in ROM analysis. Still, we advise to ensure adequate leg fixation and consecutive acquisition of the pulse sequences used for torsion measurement. Therefore, in the course of our study, we developed a special splint to immobilise the feet and thus minimise leg movement during MRI examinations for even more reliable measurements in the future. Overall, the difference in femoral version between CT and MRI was 0.35° ± 3.12° (− 6.3° to 6.5°).

A further limitation of our study is that only pathological angles obtained by ROM simulation using imaging data of patients were available, but no reference values from normal individuals. We do not have detailed information on the interanalyst variation of the technicians who performed manual segmentation. Another limitation of our study is the small number of patients.

However, the mean difference in impingement detected by CT- and MRI-based ROM analysis of − 0.1° ± 2.4° (− 21° to 13°) indicates that MRI-based ROM analysis is sufficiently accurate. There was a 97% match of ROM findings between CT and MRI. In 21.6% of the cases an abnormal ROM was detected.

Omission of CT can reduce total costs; however, MRI is the more expensive modality. The extended MRI protocol used in our study is only 4 min longer than our routine pelvic MRI protocol. However, general contraindications to MRI apply. A diagnostic CT scan of the pelvis exposes the gonads to an ionizing radiation dose of 0.02 Gy, increasing the risk of hereditable diseases by 5% [17].

More studies are needed to further investigate the performance of semiautomatic ROM analysis based on CT and MRI and the potential for fully automated ROM measurement. These studies should include more patients with different types of hip deformities. Moreover, they should address the influence of soft tissue on results. In particular, the integration of the labrum as another stabilising component of the hip will be another important step in the advancement of virtual ROM analysis. MRI-based virtual planning of correction osteotomies and virtual ROM analysis for assessing the outcome of surgery are possible future clinical applications. Another promising application to be investigated is the feasibility of semiautomatic and automatic virtual ROM analysis of MR images obtained in children.

## Conclusion

Overall, on most measures, MRI-based semiautomatic analysis of hip morphology and virtual ROM analysis is equivalent to CT. Our findings suggest that, in patients with hip deformities, an MRI alone, with its unique capability to simultaneously image soft tissue pathology (e.g., related to cartilage and the labrum) and bony contours, would suffice in the preoperative analysis of ROM and hip morphology. A separate CT scan with its consequent cost and radiation exposure would not be required and should be avoided, especially when examining younger individuals.
